# Porcine Circovirus Type 2 Rep Enhances IL-10 Production in Macrophages via Activation of p38-MAPK Pathway

**DOI:** 10.3390/v11121141

**Published:** 2019-12-10

**Authors:** Xingchen Wu, Xiaoya Wang, Tengfei Shi, Le Luo, Dan Qiao, Zhenyu Wang, Cong Han, Qian Du, Dewen Tong, Yong Huang

**Affiliations:** College of Veterinary Medicine, Northwest A&F University, Yangling 712100, China; xingchenwu2019@163.com (X.W.); wangxiaoya259@163.com (X.W.); dewey3600@nwsuaf.edu.cn (Q.D.)

**Keywords:** porcine circovirus type 2 (PCV2), interleukin-10 (IL-10), Rep, p38-MAPK pathway, thymine DNA glycosylase (TDG)

## Abstract

Porcine circovirus type 2 (PCV2) is one of the major threats to pig farms worldwide. Although PCV2 has been identified to promote IL-10 production, the detailed regulatory roles of PCV2 Rep for IL-10 production remain unclear. Herein, we first found that PCV2 Rep, rather than PCV1 Rep, enhanced IL-10 expression at the later phase of PCV2 infection in porcine alveolar macrophages (PAMs). Furthermore, we found that PCV2 Rep directly activated the p38-MAPK pathway to promote transcription factors NF-κB p50 and Sp1 binding to the *il10* promoter, but PCV1 Rep did not. During PCV2 infection, however, PCV2 Rep promoted the binding activities of NF-κB p50 and Sp1 with the *il10* promoter only at the later phase of PCV2 infection, since Rep proteins only expressed at the later phase of the infection. Moreover, silence of the thymine DNA glycosylase (TDG), a Rep-binding protein, significantly reduced the binding activities of NF-κB p50 and Sp1 with *il10* promoter, resulting in the reduction of IL-10 production in PCV2-inoculated PAMs at the later phase of infection. Taken together, our results demonstrate that Rep proteins enhance IL-10 production during PCV2 infection of PAMs via activation of p38-MAPK pathways, in which host TDG is a critical mediator.

## 1. Introduction

Porcine circovirus type 2 (PCV2) aggressively spreads throughout the world and seriously hinders the economic development of the pig industry worldwide [1,2]. As an immunosuppressive pathogen, PCV2 infection can increase the risk of porcine reproductive and respiratory syndrome virus (PRRSV), porcine parvovirus (PPV), and other viruses or bacteria infection, leading to porcine circovirus associated diseases (PCVAD) [3].

So far, among the potential 11 open reading frames (ORFs) of the PCV2 genome sequence, only four ORFs have been studied in-depth, whose encoding proteins are currently recognized as the major functional and structural proteins [4]. As one of the two largest genes in the PCV genome sequence, ORF1 encodes a 35.8 kDa virus replication-associated protein (Rep), which is considered to be necessary for viral replication and plays a vital role in cell-mediated immunity [5,6]. Although ORF1 is highly conserved between PCV1 and PCV2, they may play different roles in the virus-induced immune response. Interleukin-10 (IL-10), originally identified as an inhibitor of interferon-gamma (IFN-γ) and Interleukin-2 synthesis in Th2 cells [7], efficiently inhibits proliferative and cytokine responses in T cells and has shown to mediate both immunological unresponsiveness and the suppression of immune reactions [8,9]. As a key regulatory anti-inflammatory cytokine of multiple immune cells, IL-10 plays a pivotal role in limiting excessive inflammatory responses [10]. In the context of infectious disease, studies have reported an increase in pathogen clearance in the absence of IL-10 [11,12]. Our previous study has revealed that PCV2 infection promotes IL-10 expression, and mitogen-activated protein kinases (MAPKs) and phosphoinositide 3 kinase PI3K/Akt signaling pathways are involved in the regulation of IL-10 production in porcine alveolar macrophages (PAMs) during PCV2 infection [13]. Meanwhile, we observed that the Rep has also played roles in IL-10 expression during PCV2 infection. Up to date, the exact role that Rep plays in PCV2-induced IL-10 secretion is still unclear.

Herein, we first confirmed that PCV2 Rep enhances the production of IL-10 in PCV2-infected-PAMs at the later phase. Then, we explored the roles of the NF-κB, PI3K/Akt, ERK, and p38-MAPK signaling pathways in IL-10 expression induced by PCV2 Rep in PAMs, and further identified the regulatory roles of transcription factors NF-κB p50 and Sp1 in promoting the IL-10 transcription. Then, we figured out the function of Rep binding protein TDG in the regulation of transcription factor (NF-κB p50 and Sp1) activities and IL-10 expression. These results provide new insight for understanding how PCV2 Rep enhances IL-10 expression during PCV2 infection.

## 2. Materials and Methods

### 2.1. Cells and Viruses

The porcine alveolar macrophage cell line (CRL-2843) and human embryonic kidney 293A cell line (CRL-1573) were purchased from American Type Tissue Culture (ATCC, Manassas, VA, USA); PK-15 (Porcine kidney 15 cell line) were donated from the Innovative team of animal pathogen surveillance and epidemiology in Harbin Veterinary Research Institute, CAAS [14]. HEK-293A cells and PK-15 cells were maintained in Dulbecco’s minimum essential medium (12100046; Invitrogen Carlsbad, CA, USA) supplemented with 10% heat-inactivated fetal bovine serum (FBS). PAMs were maintained in RPMI 1640 medium (31800022; Invitrogen) with 10% heat-inactivated FBS (13011-8611; Tianhang Biotechnology, Huzhou, China), sodium pyruvate, nonessential amino acids, 100 U/mL penicillin, and 0.1 mg/mL streptomycin. All cell lines were plated in a fully humidified atmosphere containing 5% CO_2_ at 37 °C. Cells in the exponential phase of growth were used in our study.

PCV2-Rep1 and PCV1-Rep2 were generated from PCV1 (AY193712) and PCV2 (MH492006), which were isolated and stocked in our laboratory. Based on the study of Fenaux et al. [15], full-length PCV2 or PCV1 DNA sequences were amplified using primers F-PCVSAC and R-PCVSAC. The amplified sequences were cloned into pGEM-T Easy vector to construct the full-length PCV2 plasmid (T-PCV2) and full-length PCV1 plasmid (T-PCV1). Then, we constructed T-PCV2-Rep1 in the T-PCV2 plasmid by replacing its Rep encoding sequence by the PCV1 Rep encoding sequence using homologous recombination. Similarly, we constructed T-PCV1-Rep2 in the T-PCV1 plasmid through replacing its Rep encoding sequence by the PCV2 Rep encoding sequence. In detail, for the construction of T-PCV2-Rep1, the two fragments of the PCV2 Rep gene (Rep2A and Rep2B) in the T-PCV2 plasmid were replaced by the two fragments from PCV1 Rep gene (Rep1A and Rep1B) by homologous recombination using the ClonExpress II One Step Cloning Kit (C112–01, Vazyme, Nanjing, China); for the construction of T-PCV1-Rep2, the two fragments of the PCV1 Rep gene (Rep1A and Rep1B) in the T-PCV1 plasmid were replaced by the two fragments from the PCV2 Rep gene (Rep2A and Rep2B). The fragments Rep1A, Rep1B, Rep2A, and Rep2B were amplified by Polymerase Chain Reaction (PCR). Primer sequences were:

F-PCVSAC: GAACCGCGGGCTGGCTGAACTTTTGAAAGT;
R-PCVSAC: GCACCGCGGAAATTTCTGACAAACGTTACA;
Rep1A-F: GCATGCTCCCGGCCGCCATGGCCGCGGAACCAGGGGAAG;
Rep1A-R: ACAACCACTTCTTCACCATGGATGAATAATAAAAACCATTACGAAGTGA;
Rep1B-F: AAATTTCCGCGGATCACTAGTAGCTGAAAACGAAAGAAGTGCG;
Rep1B-R: GGCGGCCGCGAATTCACTAGTCCGCGGAGCTCCACACTC;
Rep2A-F: GCATGCTCCCGGCCGCCATGGCCGCGGGCTGGCTGAACT;
Rep2A-R: GAATTCCCGCGGCCGCCATGGAAACCATTACGATGTGATAACAAAAAA;
Rep2B-F: GGAGCTCCGCGGATCACTAGTATGCCCAGCAAGAAGAATGG;
Rep2B-R: GGCGGCCGCGAATTCACTAGTCCGCGGAAATTTCTGACAAA.

To gain recombinant PCV2-Rep1 and PCV1-Rep2 viruses, the T-PCV2-Rep1 and T-PCV1-Rep2 plasmids were digested with restriction endonuclease *Sac* II, the fragments were collected and then re-cyclized overnight using the T4 ligase, respectively. Subsequently, cyclized PCV2-Rep1 DNA or cyclized PCV1-Rep2 DNA were transfected into PK-15 cells using lipofectamine 2000 (Invitrogen) according to the manufacturer’s instructions. The transfected cells were cultured for three days, followed by frozen and thawing three times before centrifugation, and then the supernatants were collected to infect other cells and continuously propagated in PK-15 cells for at least five passages; the recombinant viruses PCV2-Rep1 and PCV1-Rep2 were obtained from the culture and purified by density gradient ultracentrifugation, and the details of the transfection, infection, and viral purification procedures were similar to those previously reported [16,17]. The copy numbers of the viruses were measured by the method as previously described [18].

### 2.2. Construction of Recombinant Adenoviruses

Rep1 from the PCV1 genome sequence and Rep2 from the PCV2 genome sequence were amplified and cloned into recombinant adenovirus vector pShuttle-CMV. The pShuttle-ORFs were recombined with the backbone vector pAdeasy-1 in *E. coli* BJ5183 and then transfected into HEK-293A cells after linearization to generate recombinant adenoviruses, according to the manufacturer’s instructions.

### 2.3. Enzyme Linked Immunosorbent Assay (ELISA)

Porcine alveolar macrophages (PAMs) adhered to six well plates, and then the cells were infected with five MOI PCVs or 100 MOI rAds. In order to detect IL-10 secretion in the PCV1, PCV2, PCV2-Rep1, or PCV1-Rep2 infected cells, we harvested the culture supernatants at 24 h, 48 h, and 72 h p.i., which were replaced into fresh media at each time point; in order to detect IL-10 secretion in the rAd-Blank, rAd-Rep1, and rAd-Rep2 infected cells, cells were respectively infected with these recombinant adenoviruses for 12 h, 24 h, or 48 h, then the culture supernatants were collected for ELISA detection at indicated time points without media refreshment. The levels of IL-10 secretion were measured using a commercial ELISA kit (P1000; R&D, Minneapolis, MN, USA), according to the manufacturer’s instructions.

### 2.4. Western Blotting

The total protein of the cells was isolated in Radio-Immunoprecipitation Assay (RIPA) Buffer with Phenylmethanesulfonyl fluoride (PMSF), according to the manufacturer’s instructions (Thermo, Rockford, IL, USA). Equivalent protein subjected to Sodium dodecyl sulfate -polyacrylamide gel electrophoresis (SDS-PAGE) analysis and transferred to polyvinylidene fluoride (PVDF) membranes (Millipore Corp, Billerica, MA, USA). After blocking with 5% non-fat milk in Tris-Buffered Saline with Tween 20 (TBST) buffer for 1 h, the membranes were incubated with the following primary antibodies at 4 °C overnight. Primary antibodies included: anti-phospho-Akt (9271; CST, Danvers, MA, USA), anti-Akt (9272; CST), anti-phospho-p38-MAPK (4511; CST), anti-p38-MAPK (8690; CST), anti-phospho-ERK, anti-ERK (4695; CST), anti-p50 (12540; CST), anti-Sp1 (9389; CST), anti-c-jun (9165; CST), anti-VG5Q (abs102222; absin, Shanghai, China), anti-TDG (ab154192; abcam, Cambridge, MA, USA), anti-ZNF265 (abs130552; absin), anti-c-Myc (13987; CST), and anti-β-actin (A00702; Genscript, Nanjing, China). Subsequently, Horseradish Peroxidase (HRP)-conjugated anti-mouse IgG (BM2002; Wuhan Boster Biotech, Wuhan, China) or anti-rabbit IgG (BA1058; Wuhan Boster Biotech) were incubated for 1 h. Western Enhanced Chemiluminescence Substrate (Bio-Rad, Hercules, CA, USA) was used for enhanced chemiluminescence detection, according to the manufacturer’s instructions.

### 2.5. Quantitative Polymerase Chain Reaction (Q-PCR)

mRNA of the cells were isolated by TRIzol reagent, according to the manufacturer’s instructions. RNA concentration and purity were measured using a NanoDrop spectrophotometer (Thermo). Reverse transcription of mRNA was performed using M-MLV reverse transcriptase (Invitrogen). mRNA levels were analyzed by a Bio-Rad IQ5 Real-Time PCR System using SYBR-green based Q-PCR with specific primers. The relative quantification of mRNA was undertaken through the ∆∆Ct method [19]. Primers sequences were IL-10-F: AATCTGCTCCAAGGTTCCCG; IL-10-R: TGAACACCATAGGGCACACC; β-actin-F: GGACTTCGAGCAGGAGATGG; β-actin-R: AGGAAGGAGGGCTGGAAGAG.

### 2.6. Luciferase Reporter Assay

The porcine *il10* promoter sequence was amplified and cloned into a pGL3 basic vector (Promega, Madison, WI, USA), according to the Banday assay [20]. PAMs were transfected with a mixture of pGL-IL-10 activity reporter plasmid and pRL-TK renilla luciferase plasmid using lipofectamine 2000 (Invitrogen). Luciferase activities were measured 24 h later using the Dual-Luciferase reporter assay (Promega), according to the manufacturer’s instructions.

### 2.7. Transfection of siRNAs

Cells were seeded overnight before transfection, allowed to reach 50% confluency by the time of transfection, and transfected with Akt siRNA, p38 siRNA, ERK siRNA, p50 siRNA, VG5Q siRNA, TDG siRNA, ZNF265 siRNA, and c-Myc siRNA (Table 1) (Sangon Biotech, Shanghai, China) using lipofectamine 2000 (Invitrogen), respectively. At 24 h post-transfection, the cells were infected with PCV2 or recombinant adenoviruses for the indicated times in each experiment.

### 2.8. Chromatin Immunoprecipitation (ChIP)

The ChIP assays proceeded following the Cold Spring Harbor Protocols. Briefly, the cells were cross-linked by formaldehyde before lysis for the nuclear. The nuclear were then further lysed by nuclei lysis buffer for the chromatin and proceeded with sonication. The chromatin was quantified and divided into 100 µg per antibody. p50, Sp1, and c-jun monoclonal antibodies and irrelevant antibodies were added to the chromatin samples overnight at 4 °C, followed by protein A(G)-agarose/salmon sperm DNA beads. Protein A(G)-agarose/salmon sperm DNA beads were added to the samples again to bind to the antibodies-chromatin compound. Then, the compound was digested by proteinase K, and extracted by phenol:chloroform to purify the nucleic acids. The nucleic acids were resuspended in H_2_O and analyzed by PCR. The specific primers for PCR are AP1-F: CCAGCTGTGGAAGCTCACAA; AP1-R: GGAACAACGGGCCATGCTTA; p50-F: TTGGAGAGGTCTAGGGAAGGG; p50-R: AGAGCTGTGCCTTCTTCGTT; Sp1-F: ACACGTGAATGGAACCCACA; Sp1-R: GAGGCTACCTCTCTCCCCTT.

### 2.9. Statistical Analysis

The data were presented as means ± SEM or means ± SD, and the results are representative of three independent experiments. Data of multiple groups were analyzed by Analysis of Variance (ANOVA) and Bonferroni post-hoc test, while comparisons between the two groups were performed by unpaired t tests. *p* values of <0.05 or <0.01 were considered as statistically significant for all analyses.

## 3. Results

### 3.1. PCV2 Rep Enhances the Production of IL-10 in PAMs Porcine Alveolar Macrophages

**Figure 1:**
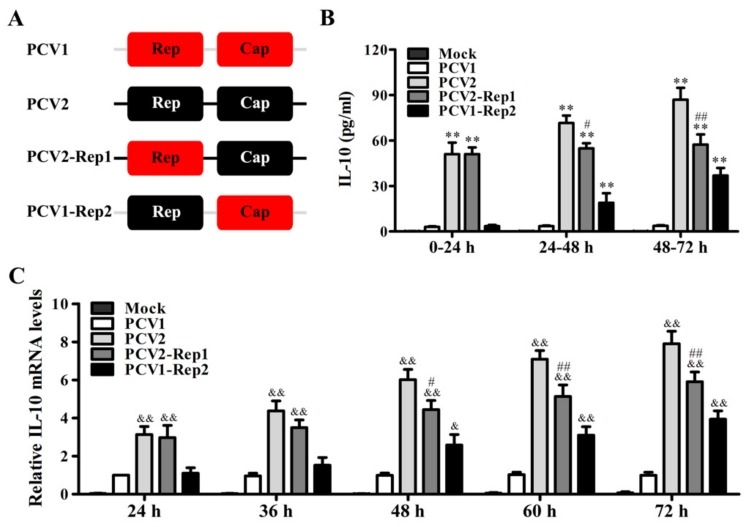
PCV2 Rep enhances the production of IL-10 in PAMs. (**A**) Model of construction of PCV1-Rep2 and PCV2-Rep1. The gray and dark lines represent the PCV1 and PCV2 backbones, respectively. (**B**) PAMs were infected with Mock, PCV1, PCV2, PCV2-Rep1, or PCV1-Rep2, and IL-10 production was analyzed by ELISA for 0–24 h, 24–48 h, and 48–72 h p.i. The columns indicate IL-10 production in each 24 h in the culture supernatants. (**C**) The IL-10 mRNA levels of the PAMs infected with Mock, PCV1, PCV2, PCV2-Rep1, or PCV1-Rep2 were detected by quantitative PCR at the indicated times post-infection. The results are mean ± SEM of three independent experiments. **P < 0.01 versus Mock infection at the same infection time point. ^#^*p* < 0.05, ^##^*p* < 0.01 versus PCV2-infected cells at the same infection time point. ^&&^*p* < 0.01 versus PCV1 infection at the same infection time point.

Previous studies have revealed that PCV2 infection induces IL-10 production in vivo and in vitro, whereas PCV1 does not show the same effect on IL-10 expression [21]. In the process of inducing IL-10 expression, PCV2 Cap plays a pivotal role in the early phase of infection [13], whether PCV2 Rep protein is also involved in the regulation of IL-10 expression during PCV2 infection remains unknown. Herein, to indicate the roles of Rep protein in regulating IL-10 expression, we used the recombinant viruses PCV1-Rep2 (a PCV1 mutant that replaced ORF1 in PCV1 backbone by PCV2 ORF1) and PCV2-Rep1 (a PCV2 mutant that replaced ORF1 in PCV2 backbone by PCV1 ORF1) to infect PAMs (Figure 1A). In 0–24 h post-inoculation (h p.i.) of these viruses, the secretion of IL-10 was barely detectable in either the PCV1- or PCV1-Rep2-infected PAMs, but was able to be detected in either PCV2- or PCV2-Rep1-infected PAMs and the IL-10 secretion showed similar levels in PCV2- and PCV2-Rep1-infected PAMs. In 24–48 h p.i., PCV1 still could not significantly induce IL-10 secretion, while PCV1-Rep2 could moderately induce IL-10 production, and the secretion of IL-10 in PCV2-Rep1-infected PAMs began to show a reduction relative to that in PCV2-infected PAMs. In 48–72 h p.i., PCV1 still could not markedly induce IL-10 production, while PCV1-Rep2-induced IL-10 secretion further increased, whereas PCV2-Rep1-induced IL-10 secretion further decreased relative to PCV2 infection (Figure 1B). Furthermore, we examined the kinetics of IL-10 mRNA in PAMs response to the infection of PCV1, PCV2, and recombinant viruses (PCV1-Rep2, PCV2-Rep1). A detailed time-course showed that starting at 24 h, the transcription of IL-10 significantly increased in the PCV2- and PCV2-Rep1-infected PAMs when compared to PCV1-infected PAMs, while the IL-10 mRNA level of PCV1-Rep2-infected PAMs began to upregulate relative to PCV1-infected PAMs from 48 h p.i. (Figure 1C). Notably, the IL-10 mRNA level of PCV2-Rep1-infected PAMs began to decrease slowly relative to PCV2-infected PAMs, after 48 h p.i. (Figure 1C). These results indicated that PCV2 Cap induces IL-10 production at the early phase of infection, and PCV2 Rep can further enhance IL-10 production at the later phase of infection in PAMs, whereas PCV1 does not induce IL-10.

### 3.2. PCV2 Rep Rather Than PCV1 Rep Directly Promotes IL-10 Production in PAMs

**Figure 2:**
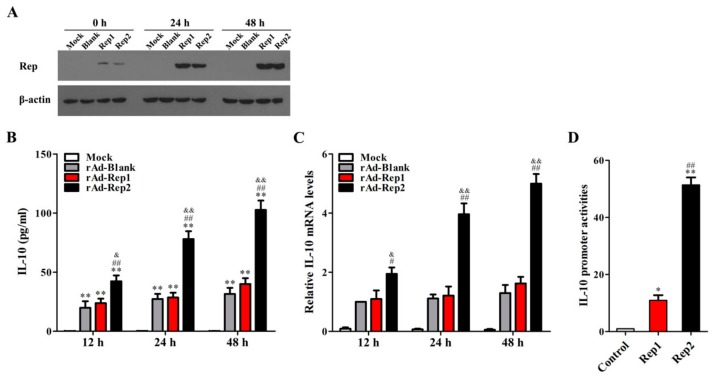
PCV2 Rep rather than PCV1 Rep directly promotes IL-10 production in PAMs. (**A**) 1 × 10^6^ of PAMs were infected with 100 MOI recombinant adenoviruses rAd-Blank, rAd-Rep1, rAd-Rep2, or Mock infection, and then the expressions of Rep were measured by western blotting. (**B**,**C**) IL-10 secretion and mRNA levels were detected by ELISA and Q-PCR, respectively. (**D**) The activities of *il10* promoter were measured in the PAMs co-transfected with the Rep1 protein expression plasmid, Rep2 protein expression plasmid, or blank vector (control) by the dual-luciferase reporter assay system. The data of (**A**) are representative of three independent experiments. The data of (**B**–**D**) are the means ± SEM of three independent experiments. *p < 0.05, **p < 0.01 versus Mock infection at same infection time point (**B**) or control (**D**). ^##^*p* < 0.01 versus rAd-Blank-infected cells at the same infection time point (**B**,**C**) or Rep1 (**D**). ^&^*p* < 0.05, ^&&^*p* < 0.01 versus rAd-Rep1-infected cells at the same infection time point.

To verify whether PCV2 Rep (Rep2) or PCV1 Rep (Rep1) could directly induce IL-10 production in PAMs, we measured the levels of IL-10 production in PAMs that were infected with adenovirus expressing Rep2 (rAd-Rep2), Rep1 (rAd-Rep1), or control blank adenovirus (rAd-Blank). The results of western blotting showed that the Rep expression increased throughout the time course of recombinant adenovirus infection and had no significant difference between rAd-Rep1- and rAd-Rep2-infected cells (Figure 2A). Compared to the uninfected cells (Mock), rAd-Blank, rAd-Rep1, and rAd-Rep2 all significantly induced IL-10 production. Compared to rAd-Blank, rAd-Rep1 did not markedly increase IL-10 production, but rAd-Rep2 dramatically increased IL-10 expression at both protein and mRNA levels (Figure 2B,C). To make clear the effects of Rep2 and Rep1 proteins on the transcription of IL-10, we detected the promoter activities of IL-10 by luciferase reporter assays in PAMs, which were separately transfected with the corresponding Rep2 or Rep1 expression plasmids. Similarly, *il10* promoter activities in Rep2-expressed PAMs were markedly higher when compared to that in Rep1-expressed PAMs (Figure 2D). These data suggest that Rep2, rather than Rep1, directly enhances the promoter activity of IL-10 to increase the IL-10 transcription.

### 3.3. PCV2 Rep Specifically Enhances the Activity of p38-MAPK Signaling Pathway to Promote IL-10 Expression in PAMs

**Figure 3:**
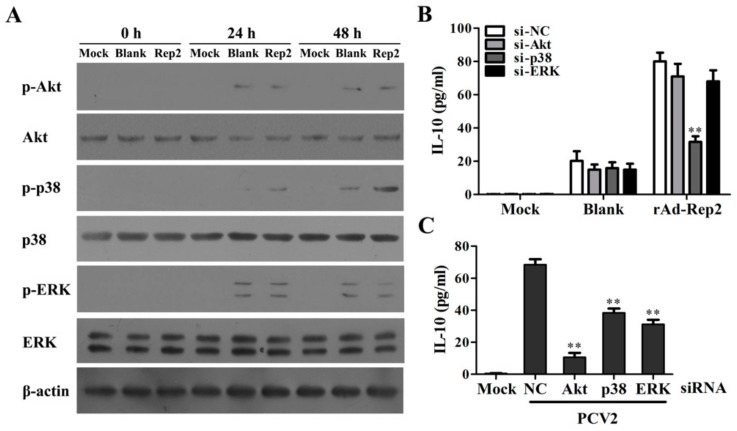
PCV2 Rep specifically enhances the activity of the p38-MAPK signaling pathway to promote IL-10 expression in PAMs. (**A**) PAMs were respectively infected with 100 MOI rAd-Blank (Blank) or rAd-Rep2 (Rep2) for 0 h, 24 h, and 48 h, and the expression and the phosphorylation levels of Akt, ERK, and p38-MAPK were detected by western blotting. (**B**) PAMs transfected with the specific siRNAs of Akt, ERK, p38-MAPK, or NC were infected with Mock, rAd-Blank, or rAd-Rep2 for 48 h, and the production of IL-10 was measured by the ELISA assay. (**C**) PAMs were transfected with the same siRNAs as B and then inoculated with 5 MOI PCV2 for 24 h. The production of IL-10 was detected by the ELISA assay. The data of (**A**) are representative of three independent experiments. The data of (**B**,**C**) are the means ± SEM of three independent experiments. **p < 0.01 versus NC siRNA-transfected cells.

Previous studies have indicated that the MAPK signaling pathways participate in the regulation of IL-10 production in macrophages [22]. To confirm whether PCV2 Rep could also activate the MAPK signaling pathways, PAMs were infected with rAd-Rep2 for 24 h and 48 h. Results showed that rAd-Blank infection could basically activate the Akt, p38-MAPK, and ERK signaling pathways relative to Mock infection. Interestingly, compared to rAd-Blank, rAd-Rep2 could significantly enhance the phosphorylation of p38-MAPK, but did not increase the phosphorylation levels of Akt or ERK (Figure 3A). Consequently, in the cells transfected with p38-MAPK specific siRNA, rAd-Rep2 infection-induced IL-10 production was markedly downregulated, whereas negative control siRNA (NC), Akt specific siRNA, and ERK specific siRNA transfection did not markedly change the IL-10 production in rAd-Rep2 infected-PAMs (Figure 3B). However, Akt specific siRNA, p38-MAPK specific siRNA, and ERK specific siRNA could all downregulate IL-10 expression in PCV2-infected cells at 24 h p.i., as per our previous report (Figure 3C). These results suggest that PCV2 Rep can upregulate IL-10 expression through enhancing the activity of the p38-MAPK signaling pathway.

### 3.4. PCV2 Rep Activates p38-MAPK Signaling to Promote NF-κB p50 and Sp1 Binding to il10 Promoter

**Figure 4:**
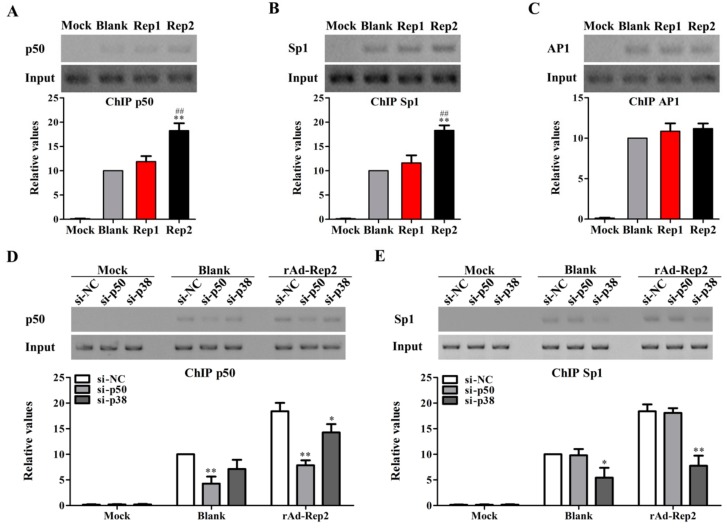
PCV2 Rep activates p38-MAPK signaling to promote NF-κB p50 and Sp1 binding to the *il10* promoter. (**A**–**C**) PAMs were infected with 100 MOI rAd-Rep1 (Rep1), rAd-Rep2 (Rep2), rAd-Blank (Blank), or Mock infection, and the binding levels of transcriptional factor NF-κB p50, Sp1, and AP1 to the *il10* promoter were detected by the ChIP assay. (**D**,**E**) PAMs were transfected with the specific siRNAs of p50, p38-MAPK, or NC, and then infected with rAd-Rep2. The binding activities of p50 and Sp1 to the *il10* promoter were detected by the ChIP assay. The data are the means ± SD of three independent experiments. *p < 0.05, **p < 0.01 versus rAd-Blank-infected cells (**A**,**B**) or NC siRNA-transfected cells in the same rAd group (**D**,**E**). ^##^*p* < 0.01 versus rAd-Rep1-infected cells.

To further investigate which transcription factors are involved in the process of Rep2-inducted IL-10 transcription, we detected the binding levels of transcriptional factors NF-κB p50, Sp1, and AP1 to the *il10* promoter in rAd-Blank-, rAd-Rep1-, or rAd-Rep2-infected PAMs by ChIP assays. The ChIP assay results showed that rAd-Blank, rAd-Rep1, and rAd-Rep2 could promote the transcriptional factors of p50, Sp1, and AP1 binding to the *il10* promoter (Figure 4A–C). Compared to rAd-Blank, rAd-Rep2 could further increase the binding levels of p50 and Sp1 to the *il10* promoter, while rAd-Rep1 could not (Figure 4A,B). However, neither rAd-Rep2 nor rAd-Rep1 could alter the binding level of AP1 to the *il10* promoter (Figure 4C). Next, we investigated which transcription factors were regulated by p38-MAPK signaling in PCV2 Rep-induced IL-10 transcription. Inhibition of p38-MAPK with specific siRNA decreased the binding levels of both p50 and Sp1 with the *il10* promoter in rAd-Rep2-infected PAMs, whereas inhibition of NF-κB activation with p50 specific siRNA only decreased the binding levels of NF-κB p50 with the *il10* promoter (Figure 4D,E). These data suggest that PCV2 Rep activates p38-MAPK signaling to promote NF-κB p50 and Sp1 binding to the *il10* promoter.

### 3.5. Rep Protein Activates p38-MAPK at the Later Phase of PCV2 Infection

**Figure 5:**
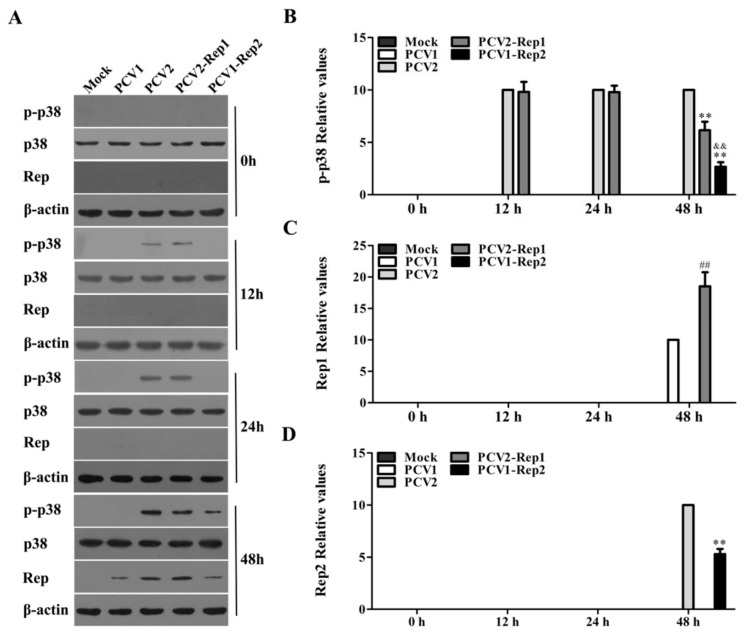
The Rep protein activates p38-MAPK at the later phase of PCV2 infection. (**A**–**D**) PAMs were infected with 5 MOI PCV1, PCV2, PCV2-Rep1, and PCV1-Rep2, and the p-p38-MAPK were detected by western blotting at 12 h, 24 h, and 48 h of post-inoculation (**A**); the relevant statistical results of p-p38-MAPK, Rep1, and Rep2 expression are shown in histograms (**B**–**D**). The data of (**A**) are representative of three independent experiments. The data of (**B**–**D**) are the means ± SD of three independent experiments. **p < 0.01 versus PCV2-inoculated cells. ^&&^*p* < 0.01 versus PCV2-Rep1-inoculated cells. ^##^*p* < 0.01 versus PCV1-inoculated cells.

To identify the characteristics of PCV2 Rep in the activation of the p38-MAPK signaling pathway, we examined the phosphorylation of p38-MAPK in cells infected with PCV1, PCV2, PCV2-Rep1, or PCV1-Rep2 at 0 h, 12 h, 24 h, and 48 h. At the earlier stage of infection (12 h and 24 h p.i.), the phosphorylation of p38-MAPK was detected in PCV2- and PCV2-Rep1-infected cells, but not in the PCV1- and PCV1-Rep2-infected cells. The levels of p-p38-MAPK did not show a significant difference in PCV2- and PCV2-Rep1-infected cells (Figure 5A,B). At the later stage of infection (48 h p.i.), p-p38-MAPK was detected in the PCV1-Rep2-infected cells, but the level of p-p38-MAPK was lower in PCV1-Rep2-infected cells than that in PCV2- and PCV2-Rep1-infected cells and the level of p-p38-MAPK in the PCV2-Rep1-infected cells was lower than that in the PCV2-infected cells (Figure 5A,B). Meanwhile, detection of the Rep proteins expression showed that either PCV1 Rep or PCV2 Rep was not able to be detected until 48 h p.i., and both Rep1 and Rep2 expression were higher in the PCV2 backbone than that in the PCV1 backbone (Figure 5A,C,D). These results suggest that the PCV2 Rep protein is involved in the regulation of p38-MAPK signaling at the later phase of PCV2 infection since Rep is expressed at the later phase of PCV2 infection.

### 3.6. Rep Protein Enhances the Binding Activities of p50 and Sp1 with il10 Promoter via p38-MAPK at the Later Phase of PCV2 Infection

**Figure 6:**
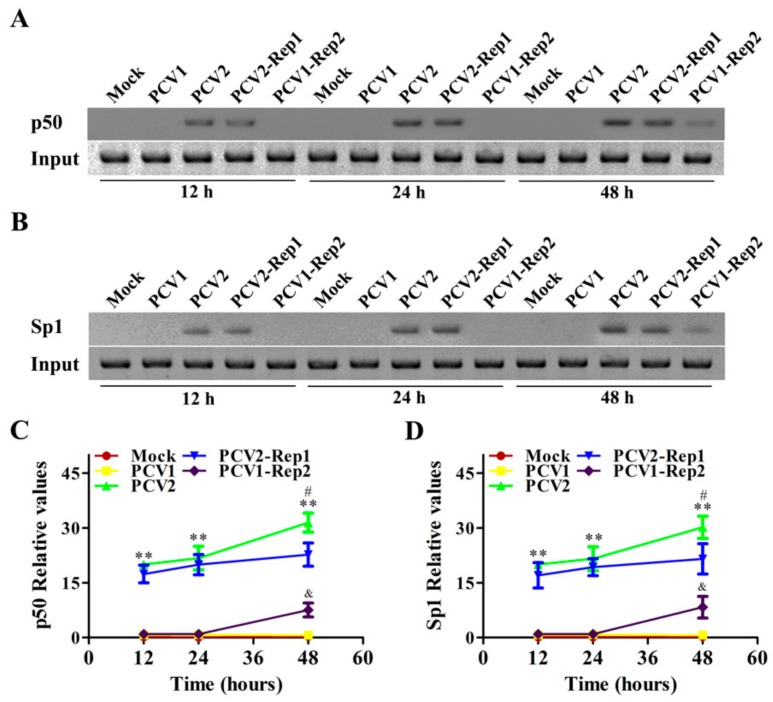
Rep protein enhances the binding activities of p50 and Sp1 with the *il10* promoter at the later phase of PCV2 infection. (**A**–**D**) PAMs were infected with 5 MOI PCV1, PCV2, PCV2-Rep1, and PCV1-Rep2, and the binding activities of p50 and Sp1 were detected at 12 h, 24 h, and 48 h using the ChIP assay (**A**,**B**); the relevant statistical results of (**A**,**B**) are shown in line graphs (**C**,**D**). The data of (**A**,**B**) are representative of three independent experiments. The data of (**C**,**D**) are the means ± SD of three independent experiments. **p < 0.01 represents the PCV2-inoculated cells versus the PCV1-Rep2-inoculated cells at the same infection time point. ^#^*p* < 0.05 represents the PCV2-inoculated cells versus the PCV2-Rep1-inoculated cells at the same infection time point. ^&^*p* < 0.05 versus PCV1-inoculated cells at the same infection time point.

Furthermore, we investigated the characteristics of binding activities between transcriptional factors (Sp1, p50) and the *il10* promoter in cells infected with PCV1, PCV2, PCV2-Rep1, or PCV1-Rep2 at 12 h, 24 h, and 48 h. At the earlier stage of infection (12 h and 24 h p.i.), the binding activity of NF-κB p50 with the *il10* promoter in PCV2-Rep1-infected cells was similar to that in the PCV2-infected cells, but was not detected in PCV1- or PCV1-Rep2-infected cells (Figure 6A,C); the binding activity of Sp1 with the *il10* promoter showed the same results (Figure 6B,D). At the later stage of infection (48 h p.i.), the binding activities of NF-κB p50 and Sp1 with the *il10* promoter in PCV2-Rep1-infected cells were lower than that in the PCV2-infected cells, whereas the binding activities of NF-κB p50 and Sp1 with the *il10* promoter in PCV1-Rep2-infected cells were beginning to be detected and appeared to be higher than that in PCV1-infected cells (Figure 6A–D). However, the binding activities of NF-κB p50 and Sp1 with the *il10* promoter in PCV1-Rep2-infected cells were still significantly lower than that in the PCV2-Rep1-infected cells and PCV2-infected cells (Figure 6A–D). These results suggest that PCV2 Rep cannot promote the binding activities of NF-κB p50 and Sp1 with the *il10* promoter at the earlier phase of PCV2 infection, but enhances the binding activities of NF-κB p50 and Sp1 with the *il10* promoter at the later phase of PCV2 infection.

### 3.7. Rep Protein Interacting with TDG Enhances the Binding Activities of Sp1 and NF-κB p50 with the il10 Promoter to Promote IL-10 Production at the Later Phase of PCV2 Infection

**Figure 7:**
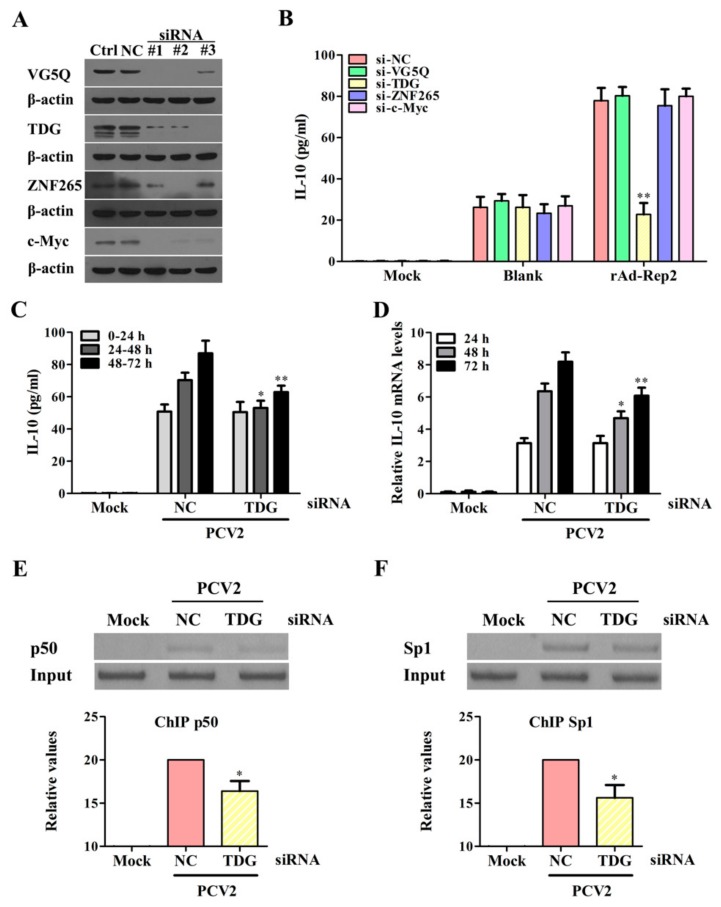
Rep protein interacts with thymine DNA glycosylase (TDG) to enhance the binding activities of Sp1 and NF-κB p50 with the *il10* promoter at the later phase of PCV2 infection. (**A**) These specific siRNAs of c-Myc, ZNF265, TDG, and VG5Q were transfected to cells for 48 h, and the efficiency of each gene silencing was detected by western blotting. (**B**) The specific siRNAs of c-Myc, ZNF265, TDG, and VG5Q siRNAs (siRNA #1 of c-Myc, siRNA #2 of ZNF265, siRNA #3 of TDG, and siRNA #2 of VG5Q) were transfected to PAMs, then rAd-Blank or rAd-Rep2 infected the cells (1 × 10^6^ cells) for 48 h. The secretion of IL-10 was measured by ELISA in different siRNA-transfected-PAMs. (**C**) The secretion of IL-10 was detected in the TDG siRNA-transfected-PAMs in 0–24 h, 24–48 h, and 48–72 h after PCV2 inoculation by ELISA. The columns indicate IL-10 production in each 24 h in the culture supernatants. (**D**) The levels of IL-10 mRNA were detected in TDG siRNA-transfected-PAMs by Q-PCR with the same incubation time points as C. (**E**,**F**) TDG siRNA-transfected-PAMs were incubated with 5 MOI PCV2, the binding activities of NF-κB p50 and Sp1 to the *il10* promoter were detected at 48 h by the ChIP assay. The data of (**A**) are representative of three independent experiments. The data of (**B**–**D**) are means ± SEM of three independent experiments. The data of (**E**,**F**) are the means ± SD of three independent experiments. *p < 0.05, **p < 0.01 versus NC siRNA-transfected cells in the same infection (**B**,**E**,**F**) or at the same time point (**C**,**D**).

Previous studies have found four binding proteins of PCV2 Rep including the transcriptional regulator c-Myc, the zinc finger protein 265 (ZNF265), thymine DNA glycosylase (TDG), and the angiogenic factor VG5Q [23]. To make clear which of them are involved in Rep mediating IL-10 induction, specific siRNAs of c-Myc, ZNF265, TDG, and VG5Q were respectively transfected into cells before rAd-Rep2 infection. The gene silencing of the targets were identified by western blotting (Figure 7A). The results showed that only the silencing of TDG could significantly decrease the production of IL-10 at 48 h post rAd-Rep2 infection, while the silencing of other Rep-binding proteins did not significantly affect IL-10 production in rAd-Rep2-infected PAMs (Figure 7B). Notably, transfection of TDG specific siRNA did not significantly decrease IL-10 production induced by PCV2 relative to the negative control siRNA in 0–24 h p.i., whereas TDG specific siRNA could decrease IL-10 secretion in 24–48 h p.i., and decrease approximately half of IL-10 secretion in PCV2-infected cells compared with NC in 48–72 h p.i. (Figure 7C). Consistently, the levels of IL-10 mRNA were also obviously decreased in the TDG-knockdown cells at the later phase of PCV2 infection (48 h and 72 h p.i.), but were not affected at the earlier phase of PCV2 infection (24 h p.i.) (Figure 7D). Furthermore, we assessed the roles of TDG in promoting the binding of NF-κB p50 and Sp1 to the *il10* promoter in PCV2-infected cells. Results showed that the binding levels of NF-κB p50 and Sp1 with the *il10* promoter in TDG-knockdown cells were significantly lower than that in negative control siRNA-transfected cells at 48 h p.i. (Figure 7E,F). These results indicate that the Rep protein interacts with host TDG to promote the binding activities of NF-κB p50 and Sp1 with the *il10* promoter that enhances IL-10 production at the later phase of infection.

## 4. Discussion

PCV2, as one of the most important swine viruses, seriously affects the development of the global swine industry [24]. Previous studies have proven that IL-10 contributes to the development of immunosuppression in some virus infection hosts [25,26]. Regardless of whether in vivo and in vitro, IL-10 expression was significantly upregulated and associated with the development of immunosuppression during PCV2 infection [27,28]. In our previous studies, we identified that the PCV2 Cap protein induces IL-10 production in PAMs through the activation of the PI3K/Akt, ERK, p38-MAPK, and NF-κB p50 signaling pathway at the earlier phase of infection [13]. In this study, we investigated the regulatory roles of PCV2 Rep protein in the induction of IL-10 in PAMs. The results demonstrate that PCV2, PCV2-Rep1, and PCV1-Rep2, but not non-pathogenic PCV1, significantly induced IL-10 production in PAMs, suggesting that PCV2 Rep, but not PCV1 Rep, can induce IL-10 expression. Further exploration found that PCV2 Rep interacting with TDG activates the p38-MAPK signaling pathway to promote NF-κB p50 and Sp1 binding to the *il10* promoter, which further increases the production of IL-10 at the later phase of PCV2 infection. All these results indicate that Rep is another critical regulator in enhancing IL-10 production during PCV2 persistent infection.

As a key anti-inflammatory cytokine, IL-10 participates in immune response and regulates by multiple signaling pathways in the activated macrophages [29,30,31]. Previous studies have shown that PCV2 infection activates the PI3K/Akt pathway to suppress premature apoptosis for improved virus growth and aggravate the effects on autophagy and PCV2 replication [32,33]. In addition, it has been reported that the p38-MAPK and ERK pathways are involved in regulating IL-10 expression [34,35,36]. Previous studies have also shown that the p38-MAPK pathway not only plays important roles in the PCV2 replication and contributes to virus-mediated changes in PK-15 cells, but is also mediated via gC1qR to suppress IL-12p40 expression to increase the risk of other pathogenic infection after PCV2 infection [37,38]. Simultaneously, it has been reported that the ERK signaling pathway is involved in PCV2 infection and is beneficial to PCV2 replication in cultured cells [39]. In the present study, we found that the PCV2 Cap could enhance expression of p-p38 in the earlier phase of PCV2 infection. PCV2 Rep was detected in PCVs-infected cells at 48 h and induced the phosphorylation of p38. These results indicate that PCV2 Rep activated the p38-MAPK signaling pathway, upregulating the production of IL-10 at the later phase of infection. Simultaneously, only the p38-MAPK signaling pathway was found to be activated by Rep to enhance IL-10 expression in the later phase of PCV2 infection. Moreover, the PCV2 Rep protein could enhance the transcription factors NF-κB p50 and Sp1 to bind with the *il10* promoter via p38-MAPK signaling activation at the later phase of PCV2 infection, aside from the earlier phase of PCV2 infection. Furthermore, we verified that specific siRNA of p38-MAPK could inhibit the upregulation of IL-10 at the later phase through inhibiting the binding activities of p50 and Sp1 to the *il10* promoter, while the specific siRNAs of ERK and PI3K/Akt did not have any influence on the production of IL-10 induced by the PCV2 Rep protein. Our results further confirmed that the PI3K/Akt, ERK, and p38-MAPK signaling pathways are also involved in regulating IL-10 expression at the earlier phase of infection as per our previous report [13]. These results demonstrate that PCV2 Rep and Cap can activate different pathways to regulate the IL-10 expression at different stages. Taken together, our results demonstrate that the p38-MAPK signaling pathway is activated by PCV2 Rep to participate in the production of IL-10 at the later phase of infection.

PCV2 is the primary causative agent of naturally occurring PMWS [40]. Previous studies have proven that PCV2 components play important roles in impairing the immune system and concurrent co-infection with other virus [41,42]. In this study, we found that PCV2 Rep could obviously promote IL-10 production, while PCV1 Rep could not. PCV2 Rep specifically activated the p38-MAPK signaling pathway to promote IL-10 production in PAMs, while PCV1 Rep could not. These data demonstrate that PCV2 Rep is another major component of PCV2 in enhancing the production of IL-10 in PCV2-inoculated cells. Meanwhile, these data also suggest that the difference in Rep protein between PCV1 and PCV2 might be another major reason for why PCV2 can induce immune suppression and cause PMWS whereas PCV1 cannot.

c-Myc, ZNF265, TDG, and VG5Q were identified to interact with Rep in PCV2-infected cells in previous studies [23,43], but the roles of these proteins in the infection and pathogenic processes of PCV2 are still unclear. In the present study, TDG was identified as an important regulator in promoting IL-10 expression. Knockdown of TDG significantly decreased the binding activities of NF-κB p50 and Sp1 to the *il10* promoter at the later phase of PCV2 infection, resulting in a dramatic reduction of IL-10 expression at both the mRNA and protein levels. These results demonstrate that the interaction of PCV2 Rep with TDG is critical for the enhancement of IL-10 expression at the later phase of PCV2 infection. However, we are still far from unveiling the complete mechanisms of PCV2 Rep regulating inflammatory cytokines, which have been addressed in our ongoing works.

## 5. Conclusions

In summary, this study provides certain evidence that PCV2 Rep can activate the p38-MAPK signaling pathway to promote NF-κB p50 and Sp1 binding to the *il10* promoter to further enhance IL-10 expression at the later phase of PCV2 infection, in which host TDG is a critical regulator. These findings might help us to understand the regulatory roles and mechanisms of PCV2 Rep proteins in boosting the production of IL-10 in porcine macrophages.

## Figures and Tables

**Table 1:** siRNA sequences for target genes used in this study.

Target Gene	Accession Number	Sequences
Akt	NM_001159776.1	AACGAGGCGAGUACAUCAAGATT
p38	XM_001929490.5	AAGCUAUCCAGACCAUUUCAATT
ERK	NM_001198922.1	AAGCACCAUUCAAGUUUGACATT
p50	KC316024.1	AAGGAGGAGAAUUACAGGUUCTT
VG5Q	XM_003123715.5	1# GCAAGACCCAUACAAGCAATT
		2# CCGUAUUUGUUCCAUGUAATT
		3# GCAGGUAACUGCCAGAUAATT
TDG	XM_021092634.1	1# GCAUAAACCUAGAUGCACUTT
		2# GGUAGAAGCGUAGUGGCCUTT
		3# GCCAGAGACUAUAGAAGACTT
ZNF265	NM_001044582.1	1# CCAGAAGAUCAGAGUGUAATT
		2# CCUAUAUUAAGGGUGCCUUTT
		3# GCCUAACGGUUCAUCUCUUTT
c-Myc	NM_001005154.1	1# CCAUGAAUUCACACUUGUUTT
		2# GCAUGAUCCAGUGCAACCUTT
		3# GCAAACUUUCCUCUGUAAATT
